# Staging Investigations in Breast Cancer: Collective Opinion of UK Breast Surgeons

**DOI:** 10.1155/2013/506172

**Published:** 2013-11-20

**Authors:** N. Chand, R. I. Cutress, R. S. Oeppen, A. Agrawal

**Affiliations:** ^1^Portsmouth Breast Care Centre, Queen Alexandra Hospital, Southwick Hill, Portsmouth PO6 3LY, UK; ^2^Breast Surgery Unit, Royal Bournemouth Hospital, Castle Lane East, Bournemouth BH7 7DW, UK; ^3^Southampton Breast Unit, Princess Anne Hospital, C Level Mailpoint 132, Coxford Road, Southampton SO16 5YA, UK

## Abstract

*Introduction*. Certain clinicopathological factors are associated with a higher likelihood of distant metastases in primary breast cancer. However, there remains inconsistency in which patients undergo formal staging for distant metastasis and the most appropriate investigation(s). *Aims*. To identify UK surgeon preferences and practice with regard to staging investigations for distant metastases. *Methods*. A survey was disseminated to members of the Association of Breast Surgery by e-mail regarding surgeon/breast unit demographics, use of staging investigations, and local policy on pre/postoperative staging investigations. Several patient scenarios were also presented. *Results*. 123 of 474 (25.9%) recipients completed the survey. Investigations routinely employed for patients diagnosed with early breast cancer included serological/haematological tests (72% respondents), axillary ultrasound (67%), liver ultrasound (2%), chest radiograph (36%), and computed tomography (CT) (1%). Three areas contributed to decisions to undertake staging by CT scan: tumour size, axillary nodal status, and plan for chemotherapy. There was widespread variation as to criteria for CT staging based on tumour size and nodal status, as well as the choice of staging investigation for the clinical scenarios presented. *Conclusions*. There remains variation in the use of staging investigations for distant disease in early breastcancer despite available guidelines.

## 1. Introduction

Accurate disease staging is important in decision-making for patients with primary breast cancer, both in treatment planning (locoregional versus systemic therapy) and in establishing the likely prognosis. Determining the presence of metastasis both at presentation and after initial treatment is a key factor in optimal diagnosis and determining ongoing treatment [1, 2]. Despite guidelines it is unclear if there is consistency as to the most appropriate initial staging investigations, and therefore the type and timing of staging investigations vary greatly between units. 

The likelihood of metastatic disease at the time of breast cancer diagnosis is very low [1], so there is no clear evidence to support universal baseline intensive staging, and in fact several studies have suggested that staging in this manner is of limited value [1–7]. The yield of staging investigations is particularly low for patients with small tumours and negative axillary nodes. However, many patients continue to undergo extensive staging at the time of diagnosis [8–10]. Overstaging can lead to unnecessary resource use (which could be better used to appropriately stage other patients), unnecessary psychological distress [11], and possible delays to treatment. 

Evidence-based guidelines aid treatment decisions and ensure quality and consistency in care [12]. Breast cancer is common, and treatment pathways therefore carry considerable resource implications. Understanding clinical practice in the staging of early breast cancer, and consideration of how well this practice mirrors available clinical guidelines is, therefore important. Several global guidelines have been produced in recent years to reduce the number of staging investigations carried out for women with early breast cancer [13–17]. 

This study aims to gain insight into the preferences and practice of surgeons and breast units in the United Kingdom with regard to staging for distant disease in breast cancer through a survey distributed to breast surgeons working within the United Kingdom. Respondents were identified through their membership in the Association of Breast Surgery (ABS), the main subspecialty professional organization covering the treatment of breast cancer in the United Kingdom and Northern Ireland. It is outside the scope of this paper to consider staging following a diagnosis of local recurrence, and so the remainder of this work focuses on staging in the setting of primary breast cancer.

## 2. Methods

An online survey was designed, containing questions pertaining to surgeon/breast unit demographics, availability and use of various staging investigations, and local policy on choice of pre/postoperative staging investigations. Several patient scenarios were also presented to determine whether consensus exists as to the choice of staging investigations in particular situations. The survey can be viewed online at http://www.surveymonkey.com/breastcancerstaging. 

Respondents were identified from the membership directory of the ABS as breast surgeons working in England, Wales, Scotland and Northern Ireland. The survey was disseminated to all members of ABS by e-mail via correspondence directly from the association. Results were collated and analyzed using Microsoft Excel 2008 for Mac and SPSS v 12.0. 

## 3. Results

**Figure 1:**
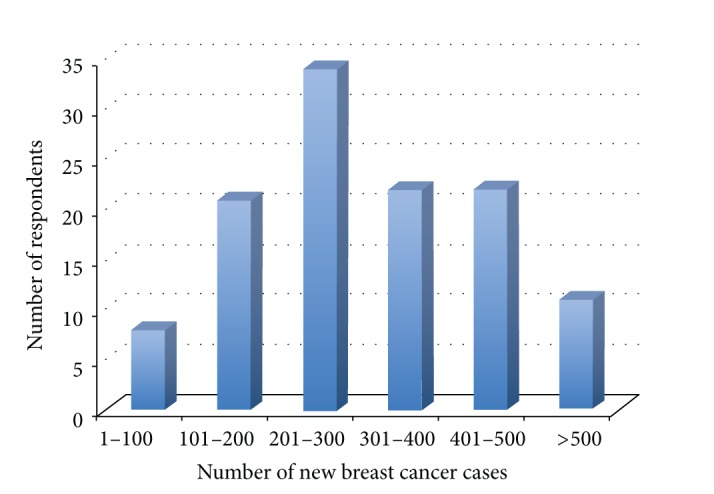
Number of New Breast Cancers seen by Respondents' Units.

Four hundred and seventy-four members listed in the ABS directory were e-mailed a link to the online survey (September, 2011). 123 recipients completed the survey (response rate 26%). From those responding a median of 3 surgeons work in each breast unit (range 1–8). Respondents were widely geographically spread within England, but underrepresented in Scotland, Wales, and Northern Ireland. The number of new cancer diagnoses seen by respondents' units broadly spanned the range of options given (Figure 1). The greatest proportion of responding units see 201–300 new cancers per year. 

Respondents were asked to indicate whether various staging modalities are either available at their unit, available at a regional hospital, or not available to them at all. The vast majority of units offer blood tests (114, 99%), CXR (114, 99%), CT (114, 99%), Magnetic resonance imaging (MRI) (110, 95.7%), and liver ultrasound (LUS) (115, 100%). Bone scintigraphy (BS) is only available at a regional hospital for 11 (10%) respondents and not available at all for 1 (1%) respondent. Positron emission tomography (PET/CT) is only available at a regional hospital for 61 (57%) respondents and not available at all for 6 (6%) respondents.

**Figure 2:**
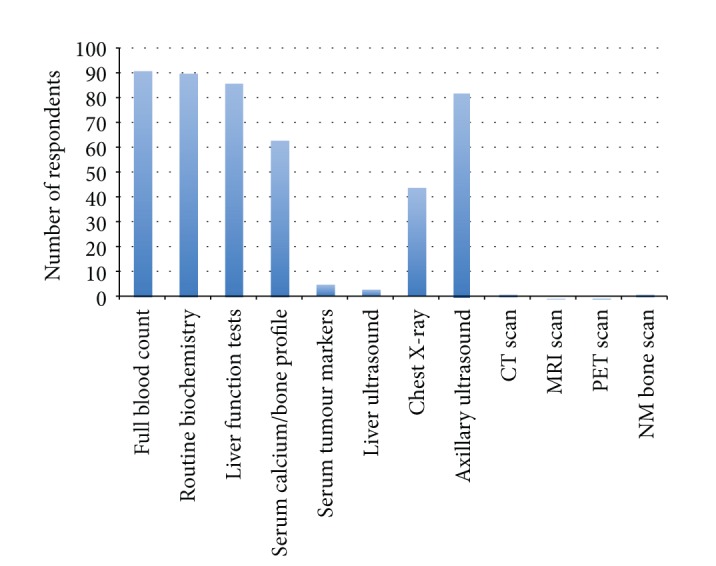
Investigations routinely performed for all patients with early breast cancer.

Respondents were asked to indicate which staging investigations are *routinely* employed for all patients diagnosed with early breast cancer (covering both staging for treatment planning, e.g., axillary ultrasound [AUS] and MRI, and staging for metastatic disease). The majority of respondents routinely use common blood tests (full blood count 91 (74%) respondents; routine biochemistry 90 (73%); liver function tests 86 (70%)). Approximately half of respondents (63, 51%) routinely use serum calcium/bone profile and 5 (4%) routinely use serum tumour markers. No respondents routinely employ MRI or PET/CT scans; only 1 respondent (1%) uses CT scanning, 1 BS, and 3 respondents (2%) use LUS routinely in early breast cancer to search for metastatic disease. CXR is used routinely by approximately one third of respondents (44, 36%) and AUS by two-thirds (82, 67%) (Figure 2). 

Respondents were then asked to describe their units' criteria for preoperative and postoperative staging for distant metastases in asymptomatic patients by CT scan for each of five themes: size, axillary nodal status, patient age, plan for neoadjuvant/adjuvant chemotherapy, and histological type of tumour. The overwhelming majority of respondents do not use patient age or histological type of tumour in decision-making for CT staging either pre- or postoperatively (96 (99%), 86 (91%) 89 (98%), and 79 (89%), resp.). 

Two-thirds (64, 67%) of respondents always perform CT staging prior to neoadjuvant chemotherapy whilst significantly fewer (17, 19%) do prior to adjuvant chemotherapy (*P* < 0.0001).

**Figure 3:**
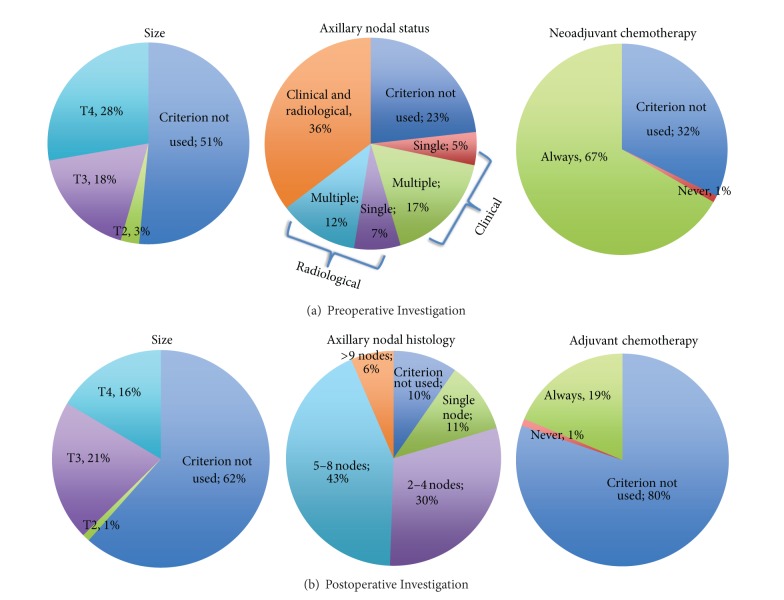
Criteria for pre- and postoperative CT staging for metastatic disease.

Tumour size and axillary nodal histology resulted in more heterogeneous responses (Figure 3). Almost half (46, 46%) of respondents would perform CT preoperatively for a T3/T4 tumour. There was no significant difference in decisions to perform CT scanning based on T-stage pre- and postoperatively (*P* = 0.33). 

The majority (85, 86%) of respondents stated that their units use CT preoperatively in the event of clinical/radiological evidence of nodal involvement. Units are much more likely to utilise CT scanning postoperatively for patients with multiple (>1) nodes than single nodal involvement (*P* = 0.0005) (Figure 3).

## 4. Clinical Scenarios

**Figure 4:**
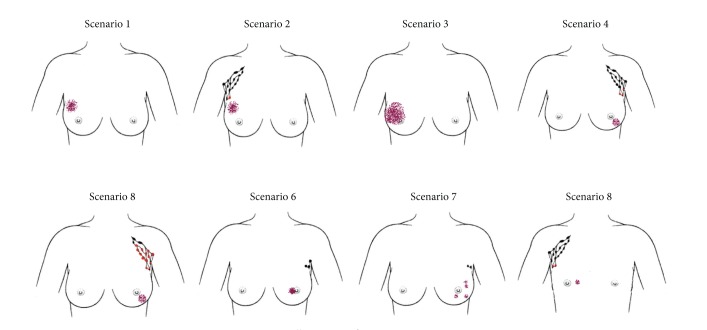
Illustrations for patient scenarios.

Respondents were given eight patient scenarios, each with a supporting illustration (Figure 4) and asked to indicate which staging investigations they would employ in each situation.

### 4.1. Scenario 1: Preoperative 43-Year-Old Female; Imaging 45 mm M5 U5; Core Biopsy; Grade 2 Invasive Ductal Carcinoma; Axillary Ultrasound Negative

83 respondents (81%) indicated that they would perform blood tests and almost half (44, 43%) a CXR. 12 respondents would perform BS and 12 (12%) CT. 5 respondents (5%) would perform LUS, whilst 1 (1%) respondent chose PET/CT. 20 respondents (19%) answered that they would not perform any staging investigations.

### 4.2. Scenario 2: Preoperative 43-Year-Old Female; Imaging 45 mm M5 U5; Core Biopsy Grade 2 Invasive Ductal Carcinoma; Axillary Ultrasound and Needle Biopsy Proven Metastatic Node

For this scenario, 13 respondents (13%) indicated that they would not perform any staging investigations. 81 respondents (81%) would perform blood tests, 42 (42%) CXR, 39 (39%) CT, 32 (32%) BS, and 4 (4%) LUS. Comparing Scenarios 1 and 2, significantly more respondents chose to undertake CT staging in a patient with confirmed axillary nodal spread (*P* = 0.0003).

### 4.3. Scenario 3: Preoperative 36-Year-Old Female; Imaging 70 mm Malignant Microcalcifications; Core Biopsy Intermediate/High Grade DCIS; Axillary Ultrasound Negative

25 respondents (25%) indicated that they would not request any staging investigations, while 75 (74%) would perform blood tests, 33 (33%) CXR, 4 (4%) CT, 4 (4%) BS, and 2 (2%) LUS. Comparing Scenarios 1 and 3, there was no significant difference in respondents' preoperative staging preferences in a patient with widespread DCIS compared with proven invasive disease in the absence of abnormal axillary nodes. 

### 4.4. Scenario 4: Postoperative 69-Year-Old Female; Histology 28 mm Grade 3 Invasive Lobular Carcinoma, 3/19 Involved Lymph Nodes

In response to this scenario, 62 respondents (63%) would request blood tests, 50 (51%) CT, 40 (41%) BS, 27 (28%) CXR, and 4 (4%) LUS. 

### 4.5. Scenario 5: Postoperative 69-Year-Old Female; Histology 28 mm Grade 3 Invasive Lobular Carcinoma, 17/19 Involved Lymph Nodes

All respondents chose to undertake some form of staging investigations. 99 respondents (98%) would request a CT, 84 (83%) BS, 67 (66%) blood tests, 25 (25%) CXR, and 6 (6%) LUS. No respondents chose PET/CT. Significantly more respondents would perform CT staging in this scenario of a patient with significant axillary nodal metastasis (98.0%), compared with Scenario 4 (40.8%) in which the patient had limited nodal disease (*P* < 0.0001).

### 4.6. Scenario 6: Postoperative 85-Year-Old Female; Histology; 20 mm Grade 2 Invasive Ductal Carcinoma, Sentinel Node Negative

Just over half of respondents (52, 52%) chose not to undertake any staging for this patient. Of the remainder, 47 (47%) chose to perform blood tests, 31 (31%) CXR, and 1 (1.0%) LUS. No respondents would perform CT, PET/CT, or BS.

### 4.7. Scenario 7: Postoperative 49-Year-Old Female; Histology Multifocal High-Grade DCIS, Sentinel Node Negative

60 respondents (59%) did not undertake any staging for this patient. Of the remainder, 40 (40%) chose blood tests, 18 (18%) CXR, 1 (1%) LUS, 1 (1%) CT, and 1 (1%) BS. No respondents chose to undertake PET/CT. Significantly fewer respondents opted to undertake postoperative staging in this scenario in which the patient has histological confirmation of in situ disease only, compared with Scenario 4 where the preoperative diagnosis is of widespread high-grade DCIS (*P* = 0.0028).

### 4.8. Scenario 8: Postoperative 62-Year-Old Male; Imaging: 14 mm Malignant Lesion; Core Biopsy Grade 3 Invasive Ductal Carcinoma; Axillary Ultrasound and Biopsy Positive for Metastasis

For this scenario, only 13 respondents (13%) chose not to undertake any staging investigations. 83 respondents (81%) chose blood tests, 40 (39%) CXR, 40 (39%) CT, 36 (35%) BS, and 4 (4%) LUS. There was no statistical difference in respondents' staging preferences for males compared with females in this scenario (Scenarios 4 and 6). 

It is evident from all of the scenarios that respondents are choosing to perform virtually no PET/CT scan at all and very few liver ultrasounds but are undertaking a significant number of bone scans. 

Respondents were asked whether their unit's staging preferences would be different if they were not subject to any financial constraints. Only 14 (11%) answered “Yes,” with several respondents commenting that CT staging should be offered for all axillary node-positive patients. Another respondent commented that PET/CT would be the main initial staging tool in the absence of financial constraints. 

Respondents were then given the opportunity to leave comments regarding staging for breast cancer. The majority of respondents commented either that they believed that routine staging for metastasis is of little value or that their unit had recently changed its practice and was performing fewer staging investigations. Comments also suggested that respondents are more likely to perform staging investigations for lobular cancers, neoadjuvant chemotherapy, preoperative clinical/radiological evidence of nodal involvement, locally invasive tumours, recurrent tumours, and inflammatory cancers. 

## 5. Discussion

Staging for distant metastatic disease in early breast cancer remains inconsistent, as illustrated by the incongruous responses given by respondents to the patient scenarios in our survey. The group responding to this survey practice in units across the United Kingdom and almost half work within units treating over three hundred new breast cancer cases per year. These results are therefore unlikely to be explained by geographical variance or caseload. Furthermore, the staging preferences of our respondents are unlikely to be significantly affected by access restrictions, as the vast majority of our respondents work within units with good availability of all staging investigations with the exception of PET/CT. 

Common sites of metastatic disease in breast cancer include bone, lung, and liver. Traditionally, staging investigations have therefore included isotope bone scan, chest radiography, and liver ultrasound. All of the staging investigations commonly employed in breast cancer (including CXR, BS, LUS as well as biochemical assays and tumour markers) have been shown to have very low detection rates when used at the time of diagnosis. Schneider et al. [7] found that the overall rate of distant metastasis was only 3.9%, and several papers have reported the detection rates of individual staging investigations. For isotope bone scan this has been published as 0.5–11% [5, 7, 18–23], for liver ultrasound 0.24–3.3% [5, 7, 20, 24], and for chest radiography 0.2–1.2% [7, 18, 20, 25, 26].

Older guidelines, such as the National Comprehensive Cancer Network [16] recommended a comprehensive baseline workup; however, several studies have found that the routine undertaking of staging investigations is unnecessary, and this is now reflected in current guidelines. A large study found that of the 80% patients diagnosed with early breast cancer undergoing baseline BS, skeletal metastasis was only detected in approximately 6%, whilst baseline CXR and LUS had even lower pick-up rates [27]. In a cohort of 781 patients evaluated by Morris et al., 34% underwent a staging bone scan, but only 14.3% patients presented with metastatic disease at any site, and the yield of bone scans was only 15.8% [28]. Importantly in this study, the majority of patients found on staging isotope bone scan to have skeletal metastases *were *symptomatic, and there was high clinical suspicion in the vast majority of the remainder. This amounted to an incidental bony metastasis rate of only 1% [28]. Morris et al. also examined the use of staging liver investigations in their cohort and found that the yield of LUS was also very low (8.8%), and again the rate of incidental hepatic metastases found at ultrasound was less than 1% [28]. In contrast, they found that the negative predictive value of normal liver function tests was 97.6%. This suggests that patients should undergo liver biochemistry routinely, particularly as they present much lower cost [28], but our survey would suggest that over 30% of respondents are not using liver function tests routinely.

Notwithstanding this evidence, our results show that traditional investigation modalities are still being used routinely in some units, particularly chest radiography. Yet only two-thirds of respondents responded to using preoperative axillary ultrasound despite clear national recommendations [13]. 

Despite the wide availability of accurate imaging techniques, many still consider clinical staging to be the most useful, not least of which because it is the most cost-effective [29, 30]. Samant and Ganguly [5] reported that most patients (84%) with radiological metastases at the time of presentation had clinical signs or symptoms suggestive of metastatic disease, a finding which has been previously reported [31]. 

Moreover, the diagnostic accuracy of the imaging modalities employed in breast cancer staging can be variable: bone scanning has been described as producing 10–15% false negative and 10–30% false positive results [9, 18, 32, 33]. Routine LUS detects many incidental benign findings with a false positive rate reported as 33–52% [1, 34]. CXR too may have a considerable false-positive rate, reported as 0–23% [34]. Furthermore, staging investigations in patients with lower stage breast cancer are proportionally more likely to give false-positive results [1]. 

Evidence-based clinical practice guidelines have the benefit of allowing clinicians to utilise the outcomes of literature efficiently after they have been systematically reviewed, appraised, and summarised. Several guidelines have been issued which contain recommendations for breast cancer staging, including the Cancer Care Ontario (CCO) [15], National Comprehensive Cancer Network (NCCN) [16], the National Institute for Clinical Excellence (NICE) [13], and the British Association of Surgical Oncology (BASO) [14].

Hogeveen et al. [12] reviewed guidelines published by the American Society of Clinical Oncology (ASCO), CCO, and NICE regarding breast cancer treatment using the Appraisal of Guidelines for Research and Evaluation (AGREE) instrument. They found the guidelines to be consistently good in terms of *scope*, *purpose*, *rigour of development*, and *clarity and presentation*. However, they suggested that certain guidelines lacked strength in *stakeholder involvement*, *applicability*, and *editorial independence*. In terms of staging investigations and follow-up, the NICE guidelines consistently received the highest scores, although the key recommendations are consistent between organizations. 

Han et al. [11] found that 55% of newly diagnosed early stage breast cancer patients underwent unnecessary investigations, which suggests that adherence to guidelines for the postoperative staging of patients with breast cancer is poor. This mirrors results from several other studies [27, 28, 35]. Improving adherence to national guidelines may well free up resources to be used in reducing time to treatment and in the staging of appropriate patients [11]. It has also been shown that staging investigations increase psychological distress [27, 35], and omitting these where unnecessary will therefore potentially lead to improved patient experience. It has been reported that simply disseminating clinical guidelines is often not effective in changing doctors' behaviour and improving patient outcomes [36]. McWhirter et al. [36] showed that requests for staging investigations in early-stage breast cancer reduced following a directed educational intervention. 

National guidelines in the United Kingdom have been described as somewhat nonspecific and open to interpretation [37]. They do, however, have in common an encouragement to limit staging to only those with locally advanced disease. The staging of patients with T4 disease or any evidence of malignant lymphadenopathy is very clear [37], however, guidance for patients outside these categories but with “locally advanced disease” is less explicit. NICE recommends only that patients with T4 tumours (stage III+) should be preoperatively staged but do not clearly advocate any one imaging modality over another [13]. They further emphasise that patients “with early breast cancer should *not* undergo staging for distant metastatic disease in the absence of symptoms” [13]. BASO clearly states that “a preoperative search for occult metastases by bone scan and liver ultrasound does not yield useful information in patients with operable primary breast cancer” and that therefore “these investigations should not normally be carried out unless the patient is symptomatic, partaking of a clinical trial, or recommended for neoadjuvant therapy” [14]. They suggest that no asymptomatic patients should be routinely preoperatively staged, other than using full blood count, routine biochemistry, and liver function tests and perhaps using plain chest X-ray according to local protocol [14]. Our results show that respondents are in fact rationalising the use of routine staging, with the vast majority of respondents only choosing to undertake blood tests and/or chest X-ray routinely, and only a handful employing any other modality in the preoperative setting (Figure 2). However, when asked to describe their criteria for staging by CT scan, over 20% respondents would stage a patient with clinically less than T4 disease (Figure 3). Whilst preoperative axillary nodal involvement is not explicitly described in either national guideline as a reason to undertake staging, it is a factor in over three-quarters of respondents' staging practice. However, it is also clear that practice is not systematic in terms of the number of involved nodes (or indeed whether they are clinically rather than radiologically apparent). 

Examining the responses to clinical scenarios further emphasises a lack of unified practice, particularly for patients with stage II breast cancer. Scenario 1 described a patient with T2 (stage IIA) disease and therefore according to national guidelines ought not to undergo any radiological staging for metastatic disease in the absence of symptoms. However, our results showed that 12% would request a bone scan and 12% CT scan. Similarly, the patients described in scenarios 2 and 4 have T2 (stage IIB) disease, and yet in Scenario 2, 39% respondents would undertake CT scan, 32% bone scan, and 4% liver ultrasound, while in Scenario 4 over half of respondents would request CT, over 40% a bone scan. Scenario 5 describes a patient with stage IIIC disease, and appropriately most respondents chose to undertake some form of radiological staging. Similarly, in Scenario 6 describing a patient with T1 (stage IA) disease, the vast majority of respondents chose not to undertake any radiological staging. These results indicate that at the extremes of disease, practice is consistent but that for those patients within the broad category of stage II disease it is much less so.

So which patients *should* undergo staging for metastasis? There is clear evidence that certain patient and tumour factors are associated with a higher likelihood of regional/distant metastatic disease in breast cancer, in particular disease stage [1, 2] (the American Joint Committee on Cancer (AJCC) staging classification for breast cancer can be accessed online [38]). Ravaioli et al. reported a prevalence of metastasis of 1.46% in low-risk patients (pT1–T3 and ≤3 positive nodes) compared with 10.68% in high-risk patients (pT4 or >3 positive nodes or pN2) as revealed by bone scan, liver ultrasound, and chest X-ray in combination [2]. They divided their cohort of 406 patients into three risk groups based on tumour and nodal stage. In their highest-risk group (T4, N1/2) the detection rate for distant metastases at staging was 15.53% leading them to recommend full staging for this group including chest X-ray, liver ultrasound, and bone scan. They went on to recommend laboratory tests and physical examination alone as adequate staging for the lowest (T1, N0-1) and moderate (T2, N0/1 or T3, N0/1) risks groups. In the cohort of 488 reviewed by Schneider et al. [7], none of the 19 patients found to have distant metastases at the time of diagnosis had tumour smaller than 1 cm, but over 18% of patients with pT4 tumours were found to have distant metastases. Bozcuk et al. [39] investigated multiple patient and pathological factors in order to identify independent predictors of distant metastasis at the time of presentation. Tumour diameter (>2 cm), lymph node involvement (1 or more) and tumour grade were statistically significant predictors of metastasis. The only others were p53 and C-erB-2 positivity, whilst several other factors including age, pathological type, and ER/PR positivity were not predictive. Based on an extensive review of the efficacy of screening investigations at demonstrating metastases in asymptomatic postoperative patients, Myers et al. [1] (for the Breast Cancer Disease Site Group of the Cancer Care Ontario Practice Guidelines Initiative) have developed a series of clear recommendations regarding screening. They concluded that bone scanning, liver ultrasound, and chest X-ray are not indicated either routinely, for in-situ/Stage I disease, or for women in whom treatment options are limited or no further treatment is possible due to age or comorbidity. They recommend bone scanning for Stage II disease and bone scanning, liver ultrasound, and chest X-ray for Stage III tumours [1]. The patients in scenarios 4 and 6 of our survey have histologically stage II disease, yet only 27.6% and 0% respondents, respectively, chose to undertake bone scanning. Similarly, the patient in Scenario 5 has stage III disease, where 83.2% respondents chose to undertake a bone scan and 24.8% a chest X-ray. Only 5.9% respondents chose to undertake liver ultrasound, however, this is mitigated by virtually all respondents choosing instead to request CT scanning. Barrett et al. reviewed over 2600 asymptomatic patients with newly diagnosed breast unit and confirmed that the incidence of occult metastasis increases with tumour stage (present in 6, 13.9, and 57% of patients with stage II (where ≥4 involved lymph nodes), III and IV disease resp.). They therefore concluded that only patients with stage III and IV disease at diagnosis require baseline staging [37]. They further suggested CT as the staging investigation of choice based on high specificity and convenience [37]. *Routine* staging CT, however, has been reported as being of little value even in poor prognostic group patients (as defined by the Nottingham Prognostic Index), with a low pickup rate and considerable rate of false positives [40].

Unfortunately, these guidelines all have the disadvantage of requiring knowledge of tumour size and definitive axillary status, which therefore precludes preoperative staging. Clinical judgement must dictate these decisions, taking into account a thorough history and clinical examination to include symptoms of metastatic disease such as bone pain. Rapidly enlarging or inflammatory tumours should prompt consideration of preoperative staging. Preoperative AUS (an important component of the NICE guideline) yields important information not only for operative planning but also regarding stage and prognosis which can inform further staging decisions, and therefore its routine use should therefore be encouraged. It may also be possible to rationalize the number of staging modalities as radiological techniques are refined. Bristow et al. investigated the concordance of CT scanning with bone scintigraphy in diagnosing bony metastasis in breast cancer. They found that 98% of patients with BS evidence of metastasis were diagnosed on CT of the thorax, abdomen, and pelvis and therefore suggested that routine BS is not required if CT is being performed [41].

So it is apparent that although guidelines do exist which address the question of staging for distant disease, responses from the questionnaire suggest that practice appears to remain inconsistent amongst UK breast surgeons. This is seen both in variation in suggested practice compared with available guidelines as well as variability in practice between surgeons in the UK. Compliance with national recommendations is reliant upon a combination of resource availability and faith in both the legitimacy of the guidelines as well as the integrity of the evidence on which they are based. 

## 6. Conclusion

Clinical and radiological staging in breast cancer is an important component of patient management. Despite national guidelines, there remains wide variation with regards to staging investigations in early breast cancer. This survey suggests that UK breast surgeons favour intensive staging of certain higher-risk subgroups.
